# Evaluating the impact of possible interobserver variability in CBCT-based soft-tissue matching using TCP/NTCP models for prostate cancer radiotherapy

**DOI:** 10.1186/s13014-022-02034-1

**Published:** 2022-04-01

**Authors:** Xiangbin Zhang, Xin Wang, Xiaoyu Li, Li Zhou, Shihong Nie, Changhu Li, Xuetao Wang, Guyu Dai, Zhonghua Deng, Renming Zhong

**Affiliations:** Division of Radiation Physics, Department of Radiotherapy, State Key Laboratory of Biotherapy and Cancer Center, West China Hospital, Sichuan University, Chengdu, 610041 People’s Republic of China

**Keywords:** Prostate cancer, Image-guided radiotherapy, Cone-beam CT, Interobserver variability, Tumor control probabilities, Normal tissue complication probabilities

## Abstract

**Background:**

Prostate alignment is subject to interobserver variability in cone-beam CT (CBCT)-based soft-tissue matching. This study aims to analyze the impact of possible interobserver variability in CBCT-based soft-tissue matching for prostate cancer radiotherapy.

**Methods:**

Retrospective data, consisting of 156 CBCT images from twelve prostate cancer patients with elective nodal irradiation were analyzed in this study. To simulate possible interobserver variability, couch shifts of 2 mm relative to the resulting patient position of prostate alignment were assumed as potential patient positions (27 possibilities). For each CBCT, the doses of the potential patient positions were re-calculated using deformable image registration-based synthetic CT. The impact of the simulated interobserver variability was evaluated using tumor control probabilities (TCPs) and normal tissue complication probabilities (NTCPs).

**Results:**

No significant differences in TCPs were found between prostate alignment and potential patient positions (0.944 ± 0.003 vs 0.945 ± 0.003, *P* = 0.117). The average NTCPs of the rectum ranged from 5.16 to 7.29 (%) among the potential patient positions and were highly influenced by the couch shift in the anterior–posterior direction. In contrast, the average NTCPs of the bladder ranged from 0.75 to 1.12 (%) among the potential patient positions and were relatively negligible.

**Conclusions:**

The NTCPs of the rectum, rather than the TCPs of the target, were highly influenced by the interobserver variability in CBCT-based soft-tissue matching. This study provides a theoretical explanation for daily CBCT-based image guidance and the prostate-rectum interface matching procedure.

*Trial registration*: Not applicable.

**Supplementary Information:**

The online version contains supplementary material available at 10.1186/s13014-022-02034-1.

## Background

As suggested by the National Comprehensive Cancer Network (NCCN) guidelines [1], the accuracy of prostate cancer radiotherapy should be verified by daily prostate alignment. The European Society for Radiotherapy and Oncology (ESTRO) guidelines recommended that prostate alignment must be based on either fiducial markers or CT-based soft-tissue matching [2]. Among these approaches, cone-beam CT (CBCT) is the most prevalent image guidance technique. However, CBCT-based soft-tissue matching is subject to interobserver variability, which is an obstacle in the standardization of prostate cancer image-guided radiotherapy (IGRT).

Several studies [3–8] have confirmed the interobserver variability in CBCT-based soft-tissue matching for prostate cancer radiotherapy. However, these previous studies primarily focused on analyzing the induced factors (e.g., observers’ experience and image quality). To date, far too little attention has been given to the impact of interobserver variability in CBCT-based soft-tissue matching.

The purpose of this paper is to analyze the impact of interobserver variability in CBCT-based soft-tissue matching, thereby providing a theoretical explanation for the standardization of prostate cancer IGRT. To simulate possible interobserver variability, couch shifts of 2 mm relative to the resulting patient position of prostate alignment were assumed as potential patient positions. The dosimetric impact of the simulated interobserver variability on targets and organs as risk (OARs) was then evaluated using tumor control probabilities (TCPs) and normal tissue complication probabilities (NTCPs).

## Methods

### Patient data acquisition

In this study, retrospective data, consisting of 156 CBCT images from twelve prostate cancer patients with elective nodal irradiation were analyzed. All patients were initially planned using volumetric-modulated arc therapy (VMAT) with prescription doses of 76 Gy for the prostate gland and seminal vesicles (CTV1) and 60.8 Gy for the pelvic lymph nodes (CTV2) in 38 daily fractions, and were replanned with a prescription dose of 16 Gy for the prostate gland only in 8 daily fractions. To enable a homogenous analysis, the patients were analyzed using the initial plans. The clinical target volume (CTV) to planned target volume (PTV) margin is 5 mm in all directions. Notably, because the anatomical changes were similar between two consecutive fractions, the CBCT images were analyzed for every three fractions.

According to our clinical setting, the slice thickness of planning CT (SOMATOM Definition AS + , Siemens Medical Solutions, Forchheim, Germany) was 3 mm. Full-arc CBCT (On-board imaging v1.4, Varian Medical Systems, Palo Alto, CA, USA) was acquired on an Edge treatment system (Varian Medical Systems, Palo Alto, CA, USA) with a slice thickness of 2 mm. The patients were required to empty their rectum and bladder, drink 500 ml of water and provide feedback on the bladder filling state prior to CT localization and treatment delivery.

### Dose reconstruction

Synthetic CT, generated by planning CT to CBCT deformable image registration in Velocity (v3.2.1, Varian Medical Systems, Palo Alto, CA), was used for interfractional dose calculation. Then, the CTV1, CTV2, rectum (whole organ), and bladder were manually delineated on CBCT and mapped to synthetic CT. Finally, the origin plan is applied to the synthetic CT to calculate the interfractional dose in the Eclipse planning system (v13.6, Varian Medical Systems, Palo Alto, CA). Because the delineation of small bowel is subject to severe streaking artifact of CBCT images, the impact of interobserver variability on the small bowel was not included in this study. To avoid interobserver variability in delineations, the targets and OARs on planning CT and CBCT were contoured by the same radiation oncologist.

### Possible interobserver variability simulation

Prostate alignment visually aligns the prostate on planning CT and CBCT. In this study, prostate alignment was performed and recorded in the Mosaiq system by an experienced radiation therapist. To simulate possible interobserver variability, combinations of 0- and 2-mm couch shifts in left–right (LR), superior-inferior (SI), and anterior–posterior (AP) directions relative to the resulting patient position of prostate alignment were assumed as potential patient positions. Consequently, a total of 27 potential patient positions were determined for each CBCT. Notably, we re-calculated the dose using the origin plan for every couch shift. The couch shifts relative to the resulting patient position of prostate alignment are listed in Table 1.

**Table 1:** The couch shifts relative to the resulting patient position of prostate alignment. LR refers to left–right, SI refers to superior-inferior, and AP refers to anterior–posterior

Potential patient positions	LR (mm)	SI (mm)	AP (mm)
P01	− 2	− 2	− 2
P02	− 2	− 2	0
P03	− 2	− 2	2
P04	− 2	0	− 2
P05	− 2	0	0
P06	− 2	0	2
P07	− 2	2	− 2
P08	− 2	2	0
P09	− 2	2	2
P10	0	− 2	− 2
P11	0	− 2	0
P12	0	− 2	2
P13	0	0	− 2
P14	0	0	0
P15	0	0	2
P16	0	2	− 2
P17	0	2	0
P18	0	2	2
P19	2	− 2	0
P20	2	− 2	− 2
P21	2	− 2	0
P22	2	0	2
P23	2	0	− 2
P24	2	0	0
P25	2	2	2
P26	2	2	− 2
P27	2	2	0

### TCPs and NTCPs

The TCPs and NTCPs were calculated using the linear-quadratic Poisson model and the Lyman-Kutcher-Burman model, respectively. These calculations were performed using the open-sourced pyradiobiology package [9] and the physical dose exported from the Eclipse planning system. The radiobiology parameters and endpoints used for TCP/NTCP calculations were specified in previous studies [10–12] and listed in Table 2.

**Table 2:** The radiobiology parameters for TCP/NTCP calculation

ROI	TCD_50_/TD_50_	γ	m	n	α/β
Prostate	38.39	0.74	–	–	1.2
Bladder	80	–	0.11	0.5	8.0
Rectum	80	–	0.15	0.12	3.9

### Data analysis

The volumes of the rectum and bladder were calculated in the Eclipse planning system. The impact of the simulated interobserver variability on the targets was evaluated using TCPs and the V_95_, D_98_, D_2_, and mean doses of CTV1 and CTV2. The impact of the simulated interobserver variability on the OARs was evaluated using NTCPs and the V_50_, V_60_, and V_70_ of the rectum and bladder. Moreover, an independent sample t-test was performed to compare the resulting TCPs, one-way analysis of variance (ANOVA) was performed to compare the resulting NTCPs using the open-sourced statsmodels packages [13], and *P* < 0.05 was regarded as statistically significant.

## Results

### The CTV volume changes and the rectal and bladder filling state

As shown in Table 3, the volumes of CTV1 and CTV2 were 56.63 ± 33.09 and 398.95 ± 118.91 (cc), respectively. The average volume changes of CTV1 ranged from − 12.80 to 6.80 (%), and those of CTV2 ranged from − 3.20 to 2.58 (%). The absolute and relative volume variations of the rectum and bladder were − 4.9 ± 13.3/9.0 ± 61.0 (cc) and − 6.1 ± 14.0/15.7 ± 31.4 (%), respectively (Additional file 1). These results indicate that the patients complied with the rectal emptying and bladder filling protocol.

**Table 3:** The volume changes of CTV1 and CTV2

Patient #	CTV1		CTV2	
	Volume (cc)	Change (%)	Volume (cc)	Change (%)
01	29.57	5.86 ± 4.90	372.93	− 2.22 ± 3.52
02	38.36	− 2.61 ± 3.85	249.89	− 1.01 ± 2.80
03	43.22	− 2.83 ± 3.61	350.52	− 1.88 ± 3.63
04	22.87	6.80 ± 10.44	362.11	2.58 ± 4.20
05	93.65	0.89 ± 2.71	413.32	− 0.73 ± 3.37
06	17.87	5.96 ± 6.15	273.21	2.36 ± 2.05
07	28.50	− 7.57 ± 7.90	363.32	0.41 ± 2.43
08	85.97	− 12.80 ± 6.44	412.31	− 1.82 ± 2.33
09	83.03	− 1.09 ± 2.90	723.73	1.13 ± 1.37
10	82.04	6.43 ± 5.77	406.29	− 1.02 ± 1.84
11	37.04	− 1.45 ± 5.50	385.01	− 3.20 ± 3.31
12	115.03	0.61 ± 6.41	474.77	0.68 ± 2.14

### The impact of the simulated interobserver variability on targets

**Fig. 1:**
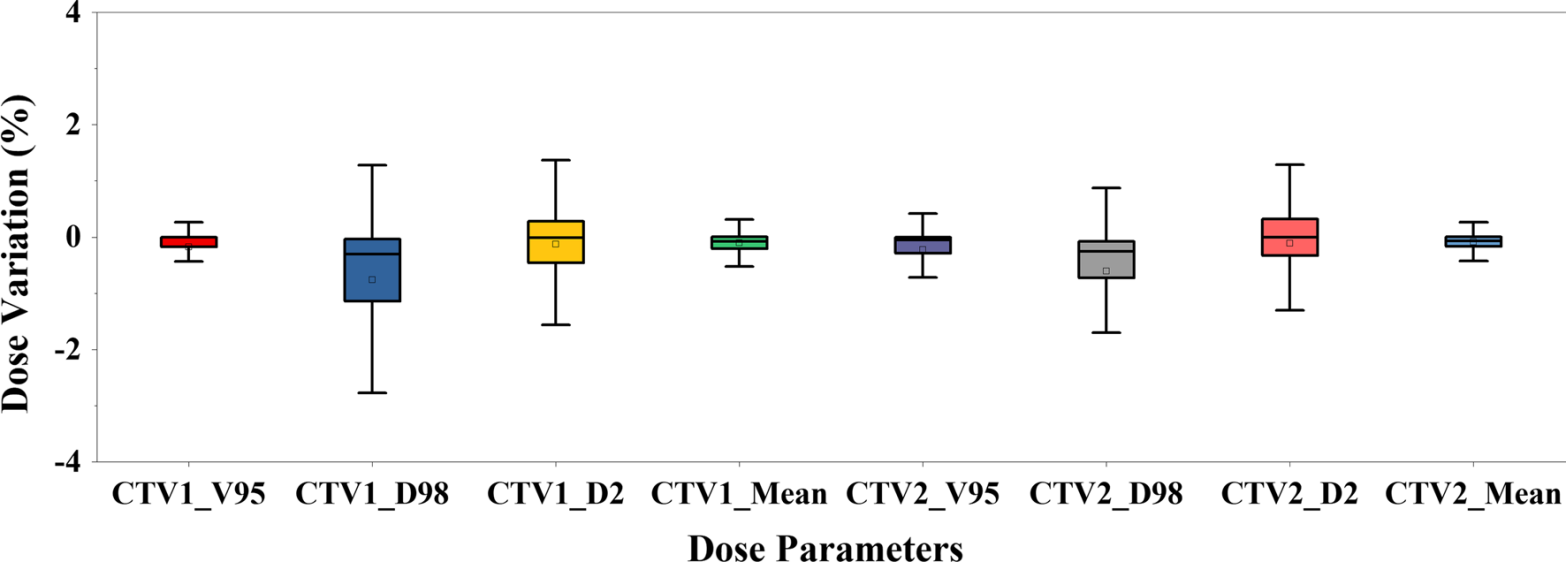
The changes in the target dosimetry (V_95_, D_98_, D_2_, and mean doses of CTV1 and CTV2) induced by the simulated interobserver variability. Error bars represent standard deviation

Compared to the resulting dose parameters of prostate alignment, the absolute deviations of the V_95_, D_98_, D_2_, and mean dose in CTV1 were 0.18 ± 0.37 (%), 0.82 ± 1.13 (%), 0.54 ± 0.54 (%), and 0.16 ± 0.13 (%), and those in CTV2 were 0.28 ± 0.52 (%), 0.67 ± 0.99 (%), 0.63 ± 0.66 (%), and 0.14 ± 0.22 (%) of the prescription doses for the 4212 potential patient positions of the 156 CBCT images, as shown in Fig. 1.

**Fig. 2:**
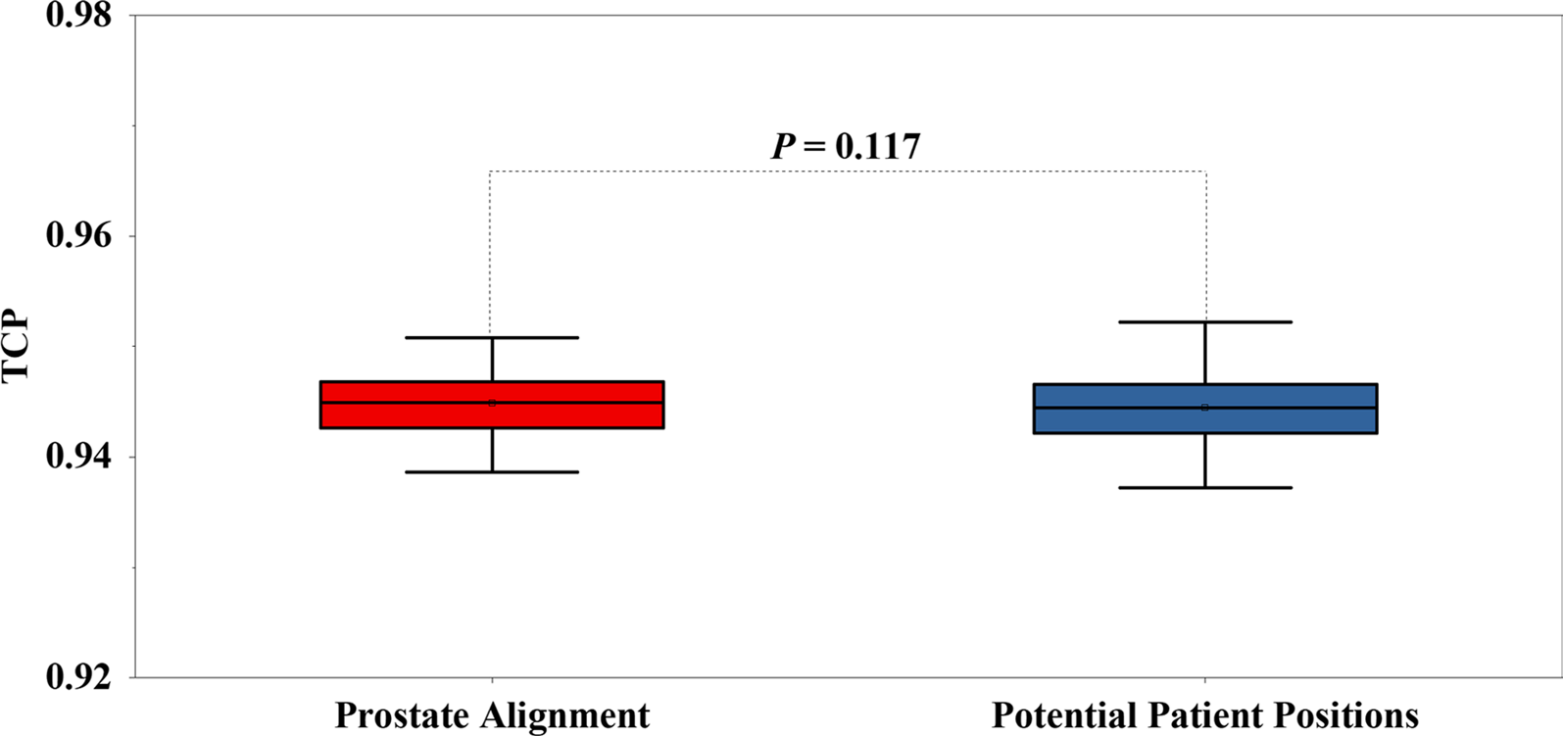
Comparison of the TCPs of CTV1 between prostate alignment and potential patient positions. Error bars represent standard deviation

Figure 2 shows that no significant differences in TCPs were found between prostate alignment and the potential patient positions, which means that the simulated interobserver variability has a minor impact on the CTV1.

### The impact of the simulated interobserver variability on OARs

**Fig. 3:**
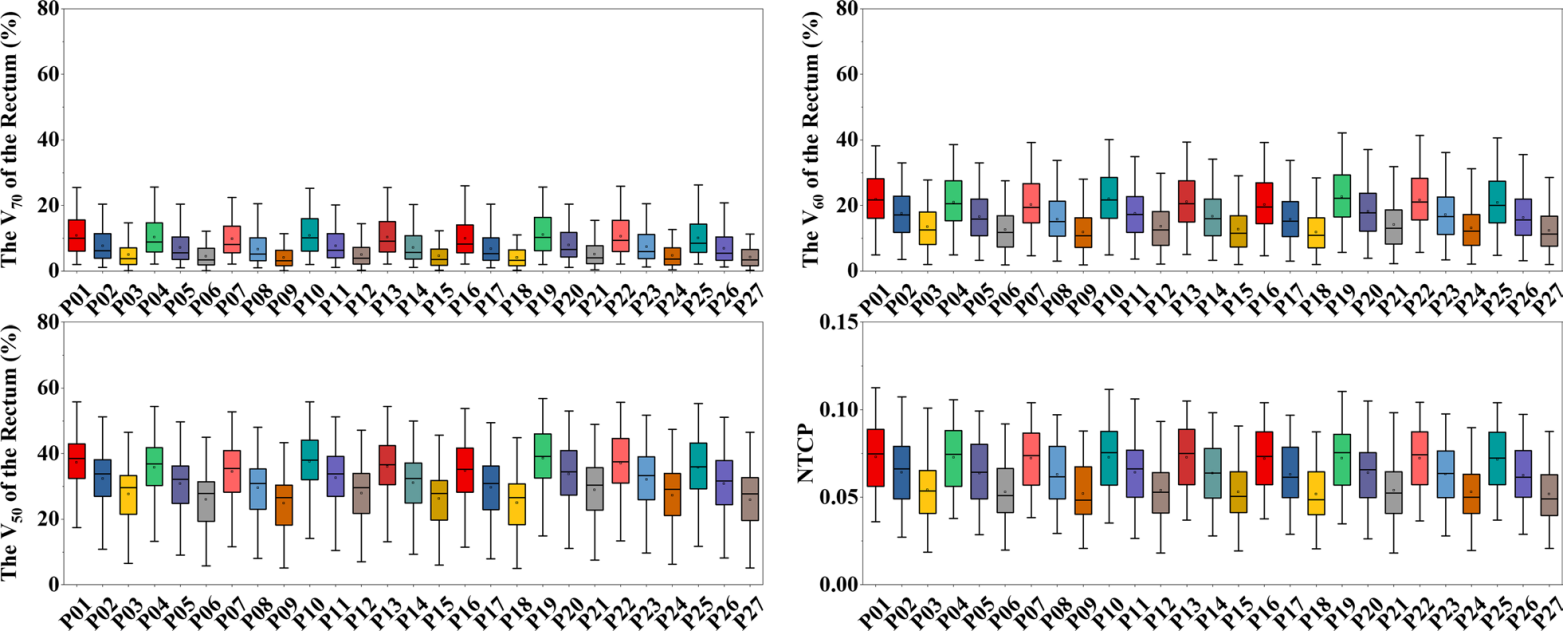
The volumetric dose parameters (V_50_, V_60_, and V_70_) and NTCPs of the rectum among the potential patient positions. Error bars represent standard deviation

As shown in Fig. 3, the volumetric dose parameters and NTCPs of the rectum were highly influenced by couch shifts in the anterior–posterior (AP) direction. On average, a 2-mm couch shift in the posterior direction will contribute to 2.88 (%), 4.18 (%), 4.84 (%), and 0.97 (%) decrease in the V_70_, V_60_, V_50_, and NTCPs of the rectum, respectively. The results of ANOVA indicate that the simulated interobserver variability has a statistical impact on the NTCPs of the rectum (*P* < 0.001).

**Fig. 4:**
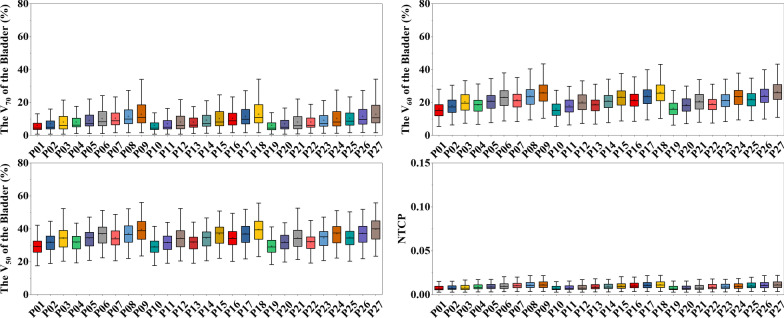
The volumetric dose parameters (V_50_, V_60_, and V_70_) and NTCPs of the bladder among the potential patient positions. Error bars represent standard deviation

In contrast, the volumetric dose parameters of the bladder were highly influenced by both the couch shifts in the AP and superior-inferior (SI) directions, as shown in Fig. 4. However, the average NTCPs of the bladder were within 1.12 (%) and relatively low. To an extent, the impact of the simulated interobserver variability on the NTCPs of the bladder is negligible.

**Fig. 5:**
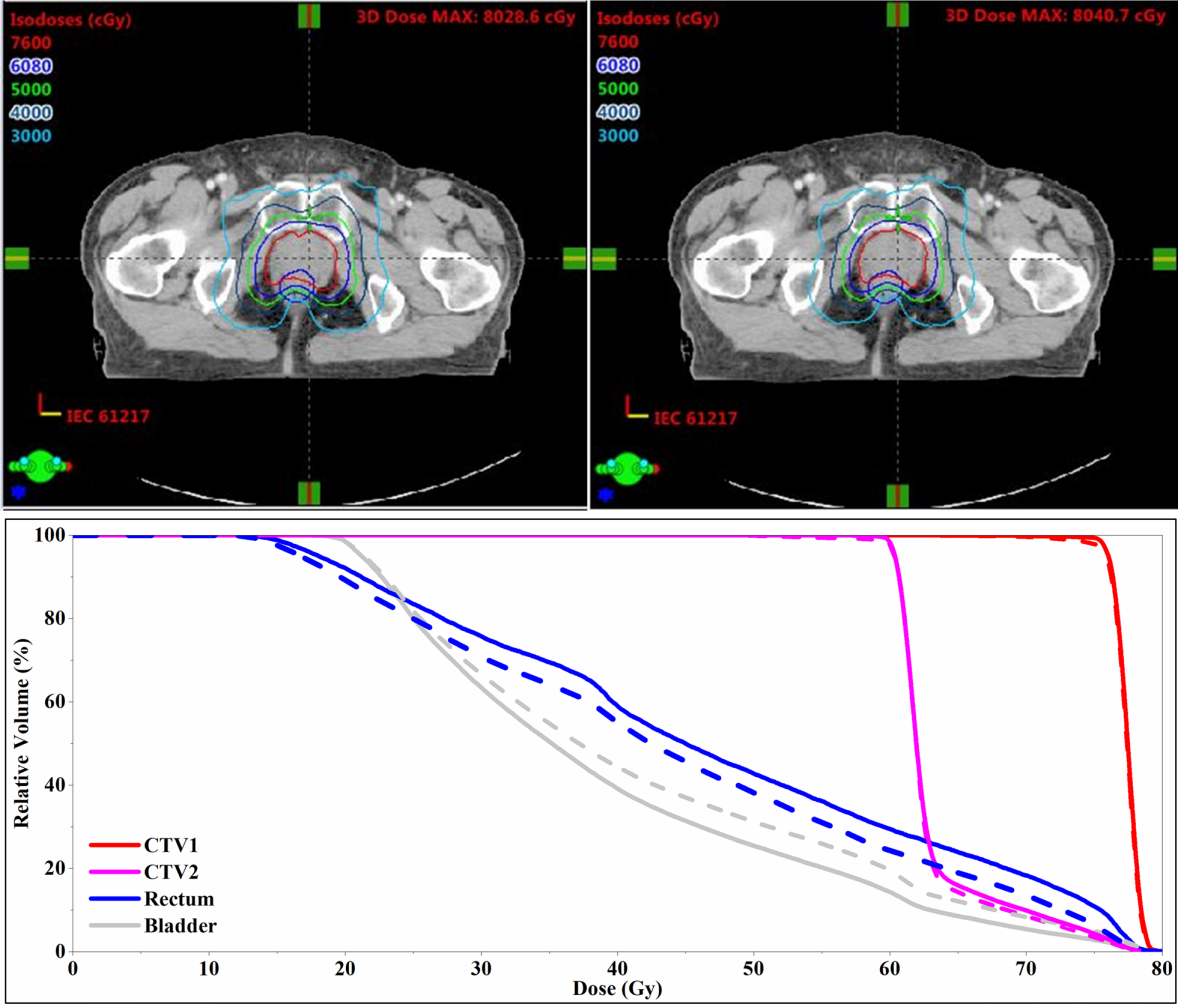
An example of interobserver variability impact on dose distribution and dose volumetric histogram. The left and right dose distribution refer to the resulting dose distribution of prostate alignment and a 2-mm couch shift in the posterior direction, respectively. The solid dose volumetric histogram refers to prostate alignment, and the dashed dose volumetric histogram refers to a 2-mm couch shift in the posterior direction

Figure 5 shows an example of interobserver variability impact on dose volumetric histogram. In this treatment fraction, the decrease in the NTCP of the rectum was 1.16 (%), while the increase in the NTCP of the bladder was only 0.19 (%), using a 2-mm couch shift in the posterior direction.

## Discussion

For the standardization of prostate cancer IGRT, it is valuable to train observers to eliminate the interobserver variability in CBCT-based soft-tissue matching. Therefore, in this study, we attempted to analyze the impact of possible interobserver variability on targets and critical OARs, thereby providing a theoretical explanation for the clinical practice of CBCT-based soft-tissue matching. Our analysis shows that only the NTCPs of the rectum were highly influenced by the simulated interobserver variability.

The TCPs of CTV1 were insensitive to simulated interobserver variability, which suggests the robustness of CBCT-based soft-tissue matching in terms of target localization. Surprisingly, this result is consistent with previous clinical trials. Napieralska et al. [14] reported that no significant differences in five-year progression-free survival were found between fiducial- and bone-based IGRT. As reported by Kotecha et al. [15] and Li et al. [16], CBCT-based soft-tissue matching is superior to fiducial-based IGRT in terms of target dosimetry, but a favorable rate of biochemical control was achievable using both IGRT techniques. This implies that a more accurate prostate target localization IGRT technique was not correlated with superior TCP.

Theoretically, various studies have demonstrated that a 3-mm PTV margin is sufficient [17, 18]. Therefore, the CTV1 dosimetry would not be deteriorated by the 2-mm interobserver variability simulation because a 5-mm PTV margin was used in this study. Furthermore, several studies [19–21] have confirmed that PTV dosimetry is not sensitive to anatomical changes. Under this condition, the factors affecting the CTV1 dosimetry are interfractional deformation and intrafractional motion. Mayyas et al. [22] reported that prostate interfractional deformations were mainly due to rectal volume variation. Hence, the rectal emptying protocol was strictly carried out in our hospital. As a result, the absolute volume variations of the rectum were − 4.9 ± 13.3 ml, negligibly small. In addition, Shelton et al. [23] verified that the intrafractional prostate motion was almost within 1 mm for the use of VMAT. This implies that a 5-mm PTV margin is sufficient to compensate for interfractional deformation, intrafractional motion, and simulated interobserver variability.

For CTV2, the ESTRO guidelines [2] recommended a 7-mm PTV margin, while a 5-mm PTV margin was used in this study. However, the changes in the CTV2 dosimetry induced by the simulated interobserver variability were similar to those in the CTV1 dosimetry (Fig. 1). A possible explanation for this is that the volumetric dose parameters in a larger target (CTV2) were less sensitive to the same couch shifts. If we used a 7-mm PTV margin, the CTV2 dosimetry would be even less affected by the simulated interobserver variability compared to the use of a 5-mm PTV margin. In addition, pelvic lymph nodes were irradiated for prophylactic purposes. Therefore, the TCPs of CTV2 were not included in this study.

Interestingly, the NTCPs of the rectum were deemed to be highly influenced by the simulated interobserver variability, while the NTCPs of the bladder were not. This result may be explained by the fact that the average NTCPs of the bladder ranged from 0.75 to 1.12 (%) among the potential patient positions. In other words, the changes in the NTCPs of the bladder were negligibly small. Moreover, this is consistent with the fact that the bladder is less radiosensitive than the rectum [11]. The volumetric dose parameters and NTCPs of the rectum were highly influenced by the couch shift in the AP direction, while those of the bladder were sensitive to the couch shifts in both the AP and SI directions (Additional file 2). This inconsistency may be due to the bladder being located in the direction superior to the high-dose PTV (PTV1).

The TCPs of the target can be effectively ensured by prostate alignment despite the interobserver variability in CBCT-based soft-tissue matching. The NTCPs of the rectum were deemed to be highly influenced by interobserver variability, while the NTCPs of the bladder were not. This provides a theoretical explanation for the prostate-rectum interface soft-tissue matching procedure [24]. More specifically, emphasis should be placed on both the rectum and the prostate in CBCT-based soft-tissue matching. In addition, compared with fiducial-based target localization, volumetric CBCT-based soft-tissue matching is superior in utilizing anatomical information for the potential use of OAR sparing. For this reason, daily IGRT is superior to weekly IGRT. However, previous clinical trials [25–29] mainly focused on analyzing the IGRT-based PTV margin reduction for OARs sparing. Therefore, detailed investigations are needed to establish the clinical protocol for a specific IGRT technique [30].

Besides, Fiandra et al. [3] reported that the average interobserver variability between senior radiation oncologists and radiation therapists in LR, SI, and AP directions were 1.32 mm, 1.69 mm, and 2.05 mm, respectively. Similarly, the results in the study of Jereczek-Fossa et al. [6] were 1.9 mm, 0.9 mm, and − 0.7 mm. Recently, Hirose et al. [5] took contour-based patient positioning as a reference, and found that the average interobserver variations among six experienced radiation therapists in LR, SI, and AP directions were 0.5, 0.9, and 0.9 mm, respectively. Therefore, in this study, maximum couch shifts of 2 mm relative to the resulting patient position of prostate alignment were used for interobserver variability simulation. Furthermore, we simulated a maximum couch shift of 5 mm (with 1 mm stepwise) in anterior and posterior directions for one treatment fraction, and found highly linearity between the NTCPs of the rectum and couch shifts in the anterior and posterior direction (Additional file 3).

The main drawback of this study is that the data from the prostate gland and the seminal vesicles were combined for analysis. As a result, the movements of the seminal vesicles independent of the prostate gland were not quantified. Therefore, in the process of CBCT-based soft-tissue matching, we ensured that the prostate gland and seminal vesicles were inside the prescription isodose line of the planned dose distribution. The results in this study showed that the CTV dosimetry and TCP were negligibly affected, although seminal vesicles move independently from the prostate. Besides, from the physical point of view, the variations of the patients without elective nodal irradiation are a subset of those of the patients with elective nodal irradiation. Thus, the conclusion obtained from the patients with elective nodal irradiation can be extrapolated to the patients without elective nodal irradiation.

## Conclusions

In conclusion, we have proposed one method to simulate interobserver variability in CBCT-based soft-tissue matching. The TCP/NTCP analysis showed that the NTCPs of the rectum, rather than the TCPs of the target, were highly influenced by the interobserver variability in CBCT-based soft-tissue matching. More specifically, the NTCPs of the rectum were highly linearly correlated with the couch shifts in the anterior–posterior direction. These provide theoretical guidance in prostate IGRT clinical practices: (1) emphasis should be placed on both the rectum and the prostate in CBCT-based soft-tissue matching; (2) compared with fiducial-based target localization, volumetric CBCT-based soft-tissue matching is superior in utilizing anatomical information for the potential use of OAR sparing; (3) daily IGRT is superior to weekly IGRT.

## Supplementary Information

**Additional file 1**. The absolute and relative volume variations of the rectum and bladder.

**Additional file 2**. The resulting P values of paired t-test for the normal tissue complication probabilities (NTCPs) of the rectum (left) and bladder (right).

**Additional file 3**. Relationships between couch shifts in the anterior and posterior direction and the TCPs of the target and the NTCPs of the rectum for one treatment fraction.

## Data Availability

The datasets used and/or analyzed during the current study are available from the corresponding author on reasonable request.

## Abbreviations

CBCT: Cone-beam CT
IGRT: Image-guided radiotherapy
OARs: Organ at risks
CTV: Clinical target volume
PTV: Planning target volume
TCP: Tumor control probability
NTCP: Normal tissue complication probability
ANOVA: One-way analysis of variance
AP: Anterior–posterior
SI: Superior-inferior
