# Supplementary material for: Evaluating the impact of possible interobserver variability in CBCT-based soft-tissue matching using TCP/NTCP models for prostate cancer radiotherapy
Source: Radiat Oncol. 2022 Apr 1;17:62. doi: 10.1186/s13014-022-02034-1 (PMC8973574; doi:10.1186/s13014-022-02034-1)
Supplement: Supplementary file 1 — Additional file 1. The absolute and relative volume variations of the rectum and bladder. [file 13014_2022_2034_MOESM1_ESM.docx]

**Supplementary Material A**

The absolute and relative volume variations of the rectum and bladder. Pt01-Pt12 refers to patients' sequence number. Error bars represent standard deviation.

**

**
