# Supplementary material for: Evaluating the impact of possible interobserver variability in CBCT-based soft-tissue matching using TCP/NTCP models for prostate cancer radiotherapy
Source: Radiat Oncol. 2022 Apr 1;17:62. doi: 10.1186/s13014-022-02034-1 (PMC8973574; doi:10.1186/s13014-022-02034-1)
Supplement: Supplementary file 2 — Additional file 2. The resulting P values of paired t-test for the normal tissue complication probabilities (NTCPs) of the rectum (left) and bladder (right). [file 13014_2022_2034_MOESM2_ESM.docx]

**Supplementary Material B**

The resulting *P* values of paired t-test for the normal tissue complication probabilities (NTCPs) of the rectum (left) and bladder (right). Notably, Black zones and white zones refer to statistically significant (*P* < 0.05) and statistically insignificant (*P* > 0.05), respectively.

**

**

The NTCPs of the rectum were highly influenced by couch shifts in the anterior-posterior direction. For example, statistically differences were found between P01 and P02, P03, P05, P06, P08, P09, P11, P12, P14, P15, P17, P18, P20, P21, P23, P24, P26, P27. Collectively, P01 and these potential patient positions were different in the anterior-posterior direction.

The NTCPs of the bladder were highly influenced by both the couch shifts in the AP and superior-inferior (SI) directions. For example, statistically differences were found between P01 and P03, P04, P05, P06, P07, P08, P09, P12, P13, P14, P15, P16, P17, P18, P21, P22, P23, P24, P25, P26, P27. Collectively, both the couch shifts in the AP and superior-inferior (SI) directions have a statistically impact on the NTCPs of the bladder. Notably, in this study, the NTCPs of the bladder were negligibly small.
