# Supplementary material for: Evaluating the impact of possible interobserver variability in CBCT-based soft-tissue matching using TCP/NTCP models for prostate cancer radiotherapy
Source: Radiat Oncol. 2022 Apr 1;17:62. doi: 10.1186/s13014-022-02034-1 (PMC8973574; doi:10.1186/s13014-022-02034-1)
Supplement: Supplementary file 3 — Additional file 3. Relationships between couch shifts in the anterior and posterior direction and the TCPs of the target and the NTCPs of the rectum for one treatment fraction. [file 13014_2022_2034_MOESM3_ESM.docx]

**Supplementary Material C**

Relationships between couch shifts in the anterior and posterior direction and the TCPs of the target and the NTCPs of the rectum for one treatment fraction.

As shown in Supplementary Material C, the NTCPs of the rectum, rather than the TCPs of the target, were highly influenced by the couch shift in anterior-posterior direction. In addition, highly linearity was found between the NTCPs of the rectum and couch shifts in the anterior and posterior direction.
